# Genetic Testing for a Patient with Suspected 3 Beta-Hydroxysteroid Dehydrogenase Deficiency: A Case of Unreported Genetic Variants

**DOI:** 10.3390/jcm11195767

**Published:** 2022-09-29

**Authors:** Elisa Menegatti, Daniele Tessaris, Alice Barinotti, Patrizia Matarazzo, Silvia Einaudi

**Affiliations:** 1 Division of Medical Genetics, Azienda Ospedaliera Città della Salute e della Scienza, University of Turin, 10126 Turin, Italy; 2 Clinical Pathology Unit, Department of Clinical and Biological Sciences, University of Turin, 10125 Turin, Italy; 3 Department of Pediatric Endocrinology, Regina Margherita Children’s Hospital, University of Turin, 10126 Turin, Italy; 4 Federazione Malattie Rare Infantili (FMRI), Regina Margherita Children’s Hospital, 10126 Torino, Italy

**Keywords:** congenital adrenal hyperplasia, 3β-hydroxysteroid dehydrogenase, case report

## Abstract

3beta-hydroxysteroid dehydrogenase type II deficiency (HSD3B2 deficiency), a rare form of congenital adrenal hyperplasia (CAH), is characterized by varying degrees of salt loss and incomplete masculinization in males and mild virilization or normal external genitalia in females. We report the case of a patient (46XY) showing salt loss and incomplete masculinization, markedly elevated levels of 17OHP (17 hydroxyprogesterone), ACTH (Adreno Cortico Tropic Hormone), testosterone and delta4androstenedione (delta4A), low levels of cortisol and absence of bone skeletal alterations that frequently characterize POR (Cytochrome P450 oxidoreductase) deficiency. Mutation analysis by Sanger sequencing of the HSD3B2 gene showed that the patient presented with a compound heterozygote for two novel variants c.370A>G p.Ser124Gly and c.308-6 G>A. The two HSD3B2 gene variants were also present in the patient’s older brother showing only incomplete masculinization. The in silico analysis revealed a probable damaging effect of c.370A>G p.Ser124Gly: residue p.Ser124 is highly conserved among species and seems to be located in the catalytic site of the enzyme, playing a pivotal role in NAD(H) binding to its substrate. Intronic c.308-6G>A variant is predicted to be likely pathogenic; the substitution seems to cause a change in the splice acceptor site located 6bp downstream of the variant. The two siblings seem to be affected by 3β-HSD2 deficiency; nevertheless, the two novel variants are likely to cause variable expressivity of the disease.

## 1. Introduction

3β-hydroxysteroid dehydrogenase (3β-HSD) is one of the key enzymes of steroid biosynthesis in adrenal glands and gonads. 3β-HSD is a 42-kDa microsomal enzyme catalysing the conversion of the hydroxyl group to a keto group on carbon 3 and the isomerisation of Δ5steroids precursors into Δ4 ketosteroids [1,2,3]. 

3β-HSD is responsible for the conversion of pregnenolone to progesterone, 17β-hydroxypregnenolone to 17β-hydroxyprogesterone, dehydroepiandrosterone (DHEA) to androstenedione and androstenediol to testosterone. 3β-HSD is encoded by two closely related genes located on chromosome 1 (1p13.1), HSD3B2 and HSD3B1, coding for type II and I isozymes, respectively. Type I isozyme (HSD3B1) is mainly expressed in the placenta and peripheral tissues, and type II isozyme (HSD3B2) is mainly expressed in the adrenal gland and gonads [4,5]. 

Deleterious mutations in the HSD3B2 gene cause classical 3β-HSD2 deficiency (OMIM # +201810), which is an extremely rare autosomal recessive form of congenital adrenal hyperplasia. 3β-HSD2 deficiency has a prevalence of 1:1,000,000 and is associated with an impairment in the steroidogenesis in both the adrenals and gonads. The effects of classic 3β-HSD2 deficiency include dysfunction in the synthesis of glucocorticoids and mineralocorticoids in adrenal glands, with an excess of Δ5-steroids, resulting in adrenal insufficiency with or without salt wasting [5,6,7,8,9].

In addition to adrenal insufficiency, gonadal function is also impaired in patients with 3β-HSD2 deficiency. Affected male individuals encounter incomplete virilization, with clinical signs ranging from mild hypospadias to genital ambiguity due to impaired testosterone biosynthesis. 46XX individuals have normal female genitalia or show moderate signs of virilization of the external genitalia with clitoromegaly. These features are linked to an overproduction of DHEA by the foetal adrenals, which in turn can be converted to testosterone by extra-adrenal 3βHSD1 [10,11,12,13,14,15,16]. 

Consequently, the presence of peripheral 3β-HSD1 activity often complicates the hormonal diagnosis of this disorder [17].

Herewith, we report the presence of 3β-HSD2 deficiency in two siblings who attended the Pediatric Endocrinology Centre of the Regina Margherita Children’s Hospital of Turin and who presented with compound heterozygote for one novel missense variant and a splicing variant in the HSD3B2 gene. We performed in silico analysis to assess the effects of the missense variant on enzyme activity and the effect of the intronic variant on splicing.

## 2. Case Report

### 2.1. Patients, Clinical Presentation and Hormonal Analysis

#### 2.1.1. Patient n.1

A full-term newborn (gestational age = 41 weeks) was born after an uneventful pregnancy by spontaneous vaginal delivery. The birth weight and length were 3330 g and 51.5 cm, respectively. Apgar scores were 9 and 9 at 1 and 5 min, respectively. This patient showed hyperpigmentation and perineal hypospadias: both testes of 2 mL were palpable in a bifid severely hyperpigmented scrotum. At the age of 3 days, they were screened for hormonal abnormalities: their tests showed markedly elevated 17OHP levels (86 ng/mL n.v. 10 ng/mL), ACTH 250 pg/mL (n.v. 8–53), cortisol 42 µg/L (n.v. 60–230), and DHEAS 1970 mcg/L (560–2360). Testosterone and delta4androstenedione (delta4A) were 1.3 ng/mL (n.v. <0.4) and 18.5 ng/mL (n.v. 0.60–5.60) respectively. Renin, aldosterone and electrolytes were within normal range (as shown in Table 1).

The karyotype was 46 XY. Given the presence of adrenal insufficiency in congenital adrenal hyperplasia, a substitutive therapy with hydrocortisone at the initial dosage of 35 mg/mg/day was started. At the age of 18 days, the patient showed some signs of salt wasting with mild hyponatremia (Na = 130 mEq/L); hence fludrocortisone 0.05 mg/day and oral NaCl supplementation of 1 g/day were added to the therapy.

We decided to test the patient for HSD3B2 gene mutations in order to investigate a possible genetic cause for the presence of salt loss and incomplete masculinization in the absence of bone skeletal alterations that are characterized more frequently in POR deficiency. A high level of testosterone (1.3 ng/mL), measured on the third day of life, should reflect maternal hormonal interference.

#### 2.1.2. Patient n.2

Patient n.2 was the older brother of patient n.1. At the time of clinical and biochemical screening, they were 2.5 years old. They were a full-term newborn (gestational age = 40 weeks), born after an uneventful pregnancy by vaginal delivery. Their weight at birth was 3110 g and their length was 51.5 cm. Their Apgar scores were 10 and 10 at 1 and 5 min, respectively. Neurological exam was normal. Neither asthenia nor other health problems were reported. Weight was 12.5 kg (−0.56 SDS), height was 89 cm (−039 SDS). They showed the same severe perineal hypospadias as the brother, supporting the need for surgical correction, with normal scrotal gonads of 2.5 mL bilaterally (pubertal stage G1PH1). Neonatal hyperpigmentation was referred by the parents. Hormonal data did not show adrenal insufficiency (details are reported in Table 1).

## 3. Materials and Methods

### 3.1. Hormonal Testing

Hormonal tests were realized as previously reported [18]. In particular, blood samples were taken in the morning between 08:00–09:00 h, following an overnight fast. Plasma ACTH levels (pg/mL) were measured in duplicate by immunoradiometric assay (Diasorin, Saluggia, Italy). The sensitivity of the assay was 1.2 pg/mL. The mean inter- and intra-assay coefficients of variation were 4.2 and 8.4%. Serum cortisol levels (μg/dL) were measured in duplicate by radioimmunoassay (Pantec, Torino, Italy). The sensitivity of the assay was 3.6 μg/dL. The mean inter- and intra-assay coefficients of variation were 9.2 and 5.8%, respectively. Serum 17α-OHP levels (ng/mL) were measured in duplicate by radioimmunoassay (RADIM, Pomezia, Italy). The sensitivity of the assay was 0.01 ng/mL. The mean inter- and intra-assay coefficients of variation were 9.0 and 8.9%, respectively. 

### 3.2. DNA Sequencing

After we obtained informed consent, total genomic DNA was purified from EDTA-collected peripheral blood leukocytes using the QIAamp DNA Blood Midi Kit (Qiagen, Milan, Italy), according to the manufacturer’s instructions.

Direct sequencing by the Sanger method of HSD3B2 exons 1–4 and exon-intron splicing junction boundaries was performed (Ref. Seq.: NM_000198; NP_000189) (Table 2).

PCR reactions were treated with exonuclease I and shrimp alkaline phosphatase (ExoSAP-IT, USB Corporation, Cleveland, OH, USA) and sequence reactions were performed by ABI Big Dye Terminator (Applied Biosystems, Foster City, CA, USA) chemistry and analysed by ABI PRISM 3130xl Genetic Analyzer (Applied Biosystems, Foster City, CA, USA).

Sequence data were analysed using Mutation Surveyor DNA variant analysis software (Softgenetic, State College, PA, USA).

### 3.3. In Silico Analysis

The following databases and integrated tools were used to predict the effect of the two mutations:

- Alamut v1.4 (http://www.interactive-biosoftware.com (accessed on 8 June 2022));
- Varsome 11.3 (https://varsome.com/ (accessed on 8 June 2022));
- Franklin by Geeox (https://help.genoox.com/en/collections/2077313-franklin-variant-interpretation (accessed on 8 June 2022)).

With respect to the missense mutation, the evaluation of the risk of pathogenicity was thus the result of the following individual tools integration:

- MetaLR;
- REVEL;
- DEOGEN2;
- FATHMM;
- M-CAP;
- MVP;
- MutPred;
- Mutation assessor;
- PROVEAN;
- SIFT4G;
- PolyPhen2.

With respect to the intronic mutation, the integrated tools included:

- SpliteSiteFinder;
- MaxEntScan;
- NNSPLICE;
- GeneSplicer.

## 4. Results

**Figure 1:**
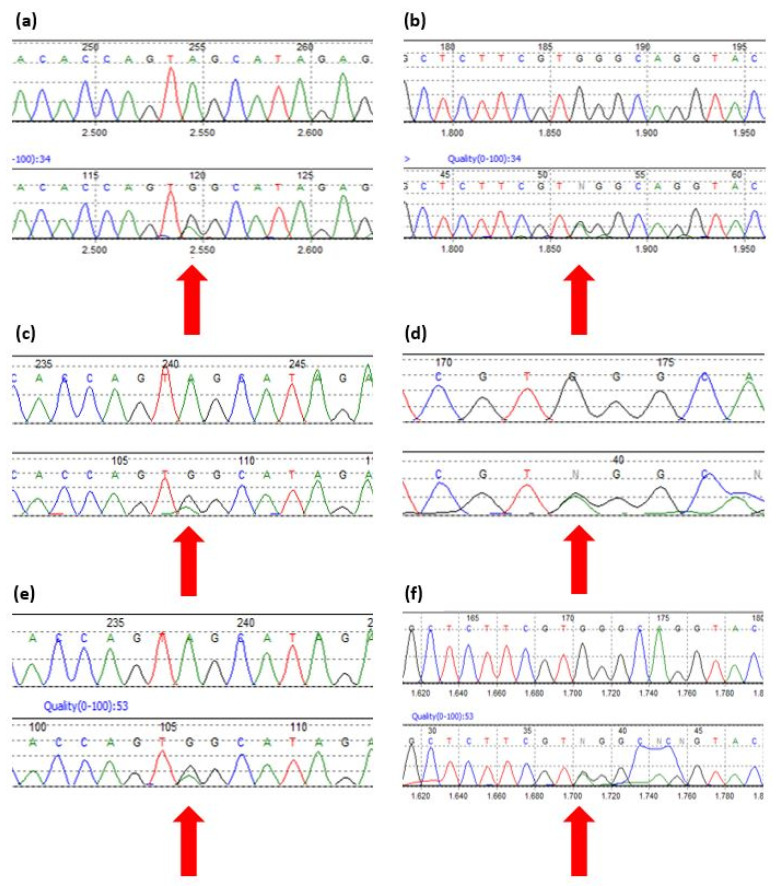
Sequences from Mutation Surveyor v3.30 reporting the mutations found in Patient n.1, Patient n.2 and their parents. (**a**) Patient n.1 missense mutation HSD3B2 c.370 A>G. (**b**) Patient n.1 intronic mutation HSD3B2 c.308-6 G>A. (**c**) Patient n.2 missense mutation HSD3B2 c.370 A>G. (**d**) Patient n.2 intronic mutation HSD3B2 c.308-6 G>A. (**e**) Father’s missense mutation HSD3B2 c.370 A>G. (**f**) Mother’s intronic mutation HSD3B2 c.308-6 G>A.

Sequencing of the HSD3B2 gene showed that Patient n.1 and their brother (Patient n.2) carried two mutations that have never been previously described: c.370A>G p.Ser124Gly and c.308-6 G>A (Figure 1a–d). Conversely, both parents were clinically healthy and heterozygous carriers of the mutations; the father carried the missense mutation, while the mother carried the intronic mutation (Figure 1e,f). 

### 4.1. HSD3B2 c.370 A>G p.Ser124Gly

The missense mutation on exon 4 c.370 A>G, leading to serine substitution in position 124 with a glycine (p.Ser124Gly), was inherited from the father and never described in the literature. 

According to all three main tools used, the variant is of uncertain significance (ACMG classification) [19]:

- Alamut: variant of uncertain significance (PM1, PM2, BP4);
- Varsome: variant of uncertain significance (PM2, PP3);
- Franklin: variant of uncertain significance, likely pathogenic (PM2, PM1 e PP2).

When focusing on each predictor and meta predictor, 11 out of 15 reported the variant as being damaging/pathogenic. Table 3 summarizes the obtained results.

### 4.2. HSD3B2 c.308-6 G>A

The variant on intron 3 (c.308-6 G>A) consists of the substitution of guanine to adenine located six nucleotides upstream from exon 4 with a frequency of 0.000004 according to gnomAD and a likely pathogenic effect (PP3, PM2) according to Varsome. When analysed using Alamut™ Visual Software (Interactive Biosoftware), three models out of four mimicking the splicing predicted a change at the acceptor site 6bp downstream to the variant:

- MaxEnt: −67.1%
- NNSPLICE: −0.9%
- SSF: −100.0%

## 5. Discussion

We reported the medical history of two siblings attending the Pediatric Endocrinology Centre of Regina Margherita Children’s Hospital of Turin who presented with two heterozygous mutations on the HSD3B2 gene that have never been previously described in the literature. Patient n.1 presented with a more severe condition, showing both an altered adrenal hormonal profile and hyperpigmentation and perineal hypospadias. On the other hand, while hormonal tests in Patient n.2 did not show adrenal insufficiency, they still presented with severe perineal hypospadias.

The first variant found in the two siblings was inherited from the healthy father and was on exon 4 (c.370 A>G), resulting in a missense mutation leading to the change of serine in position 124 to a glycine. The in silico analysis revealed a probable damaging effect of this variant: residue p.Ser124 indeed is highly conserved among species and seems to be located in the catalytic site of the enzyme, playing a pivotal role in NAD(H) binding to its substrate [20]. The first reaction during the conversion of steroid precursors is, in fact, the oxidation of the 3β-hydroxyl group to the ketone by the dehydrogenase activity, a process during which NAD+ is reduced to NADH. The intermediate Δ5,3-ketosteroid remains bound to the enzyme with nascent NADH, and the actual presence of NADH in the cofactor-binding site leads to the activation of the Δ5-Δ4-isomerase activity [5,7].

The second variant was found on intron 3 (c.308-6 G>A) and was inherited from a healthy mother. According to the ACMG recommendation, the intronic variant is classified as a VUS with the following grade: PM2 (moderate), due to the extremely low frequency in gnomAD population databases. Nonetheless, the in silico analysis revealed a likely pathogenic effect since the substitution of a guanine to an adenine seems to form a new dinucleotide resulting in a change in the splice acceptor site located 6bp downstream of the variant and consequently in a frameshift or in the activation of a new cryptic site, potentially resulting in a non-functional enzyme.

Due to the rarity of the disease, the phenotype–genotype correlation has not been fully investigated in a large case series. Albeit, in general, the functional and biochemical data are in agreement with the expressed phenotype, the clinical phenotype in the affected patients is significantly heterogeneous; moreover, identical DNA variants have been found in the HSD3B2 gene in patients showing a different clinical outcome [3,21,22]. Among others, in a recent work by Ladjouze et al., the authors report the finding of the same mutation (c.665C>A) both in a patient showing a salt-wasting form and in a patient showing a non-salt-wasting form [21].

Also, in our case, the possibility of some residual canonical splicing that may occur at some extent cannot be excluded.

Taking the above into account and also considering the role of other factors on gene expression (such as epigenetic modifications and other modifying genes), the observation of diverse clinical presentations in these two siblings sharing the same variants is not surprising [23]. 

## 6. Conclusions

We report the presence of two novel variants on the HSD3B2 gene in the compound heterozygous form that likely caused the 3βHSD2 deficiency with variable expressivity in two siblings, a missense mutation on exon 4 (c.370 A>G) and an intronic mutation on intron 3 (c.308-6 G>A), whose potential pathogenetic effect were evaluated by in-silico analysis and need functional validation. This study contributes to a better understanding of the molecular defects of 3β-HSD and of the phenotypic heterogeneity associated with this enzymatic deficit.

## Figures and Tables

**Table 1:** Details regarding the hormonal tests of patient n.1 at the age of three days and patient n.2 at the age of two and a half years.

Hormone	Patient n.1 Values	Patient n.2 Values	Normal Levels
17-OHP ng/mL	86	-	<10 (full term newborn)987654<2 (after 6 months)
ACTH pg/mL	250	53	8–53
Cortisol µg/L	42	157	60–230
Aldosterone ng/mL	514	197	70–550
PRA ng/mL/h	2.07	12.8	1.31–3.95
Dihydrotestosterone pg/mL	26	-	300–1200
Testosterone ng/mL	1.3	<0.2	<0.4
Delta4androstenedione ng/mL	18.5	-	0.60–5.60
DHEAS mcg/L	1970	779	560–2360
LH U/L	1.2	-	<1.3
FSH U/L	0.4	-	<2
Na mEq/L	136	139	136–146
K mEq/L	5.3	4.6	3.5–5.3
Cl mEq/L	105	-	97–110
Glucose	64	78	70–110

17-OHP = 17 hydroxyprogesterone (defined in the abstract). ACTH = Adreno Cortico Tropic Hormone (defined in the abstract). PRA = Plasma renin activity. DHEAS = Dehydroepiandrosterone. LH = Luteinizing hormone. FSH = Follicle-stimulating hormone. Na = Sodium. K = Potassium. Cl = Chloride.

**Table 2:** Oligonucleotides designed for the HSD3B2 mutation analysis.

Primer	Sequence
HSD3B2_1-2_FW	GCTCCAGTCCTTCCTCCAGG
HSD3B2_1-2_REV	AGGTCAACCTCCCCACACCC
HSD3B2_3_FW	GGATGTGTGACAATTCACTGC
HSD3B2_3_REV	TCTTTCTGATCCTCATTTAACCAA
HSD3B2_4_FW	CATGTGGTTGCAGCTCCTTT
HSD3B2_4_REV	GAAGAAGACAGTAAGTTGGG
HSD3B2_4INT_FW *	ACCTTGTACACTTGTGC
HSD3B2_4INT_REV *	TGTGGCGGTTGAAGGG

* Internal primers for sequencing. FW = forward. REV = reverse.

**Table 3:** A detailed summary of the predictors and meta predictors outcomes.

Predictor	Outcome
metal	Damaging (score: 0.8606)
REVEL	Pathogenic (score: 0.6129)
DEOGEN2	Damaging (score: 0.6196, 0.7482)
FATHMM	Damaging (score: −3.44)
M-CAP	Damaging (score: 0.1732)
MVP	Pathogenic (score: 0.9976)
MutPred	Pathogenic (score: 0.935)
Mutation assessor	High (score: 3.86)
PROVEAN	Damaging (score: −3.02, −3.49)
SIFT4G	Damaging (score: 0.002, 0.003)
PolyPhen2	HDivPred = probably damaging (score: 0.986)/HVarPred = possibly damaging (score: 0.898)
EIGEN	Neutral (score −0.1586)
LRT	Neutral (score 0.06347)
Mutation taster	Polymorphism (score: 0.9774)
PrimateAI	Tolerated (score 0.3522)

REVEL = rare exome variant ensemble learner. DEOGEN2 = tthis is the name. FATHMM = Functional Analysis through Hidden Markov Models. PROVEAN = Protein Variation Effect Analyzer. SIFT4G = Sort Intolerant From Tolerant version 4G. EIGEN = this is the name. LRT = this is the name. AI = the name is “Primate AI”.

## Data Availability

The contributing author can share data upon request.
